# Patterns in the Composition of Microbial Communities from a Subtropical River: Effects of Environmental, Spatial and Temporal Factors

**DOI:** 10.1371/journal.pone.0081232

**Published:** 2013-11-14

**Authors:** Lemian Liu, Jun Yang, Xiaoqing Yu, Guangjie Chen, Zheng Yu

**Affiliations:** 1 Aquatic Ecohealth Group, Key Laboratory of Urban Environment and Health, Institute of Urban Environment, Chinese Academy of Sciences, Xiamen, PR China; 2 Key Laboratory of Plateau Lake Ecology and Global Change, School of Tourism and Geography, Yunnan Normal University, Kunming, PR China; 3 University of Chinese Academy of Sciences, Beijing, PR China; Wageningen University, Netherlands

## Abstract

Microbes are key components of aquatic ecosystems and play crucial roles in global biogeochemical cycles. However, the spatiotemporal dynamics of planktonic microbial community composition in riverine ecosystems are still poorly understood. In this study, we used denaturing gradient gel electrophoresis of PCR-amplified 16S and 18S rRNA gene fragments and multivariate statistical methods to explore the spatiotemporal patterns and driving factors of planktonic bacterial and microbial eukaryotic communities in the subtropical Jiulong River, southeast China. Both bacterial and microbial eukaryotic communities varied significantly in time and were spatially structured according to upper stream, middle-lower stream and estuary. Among all the environmental factors measured, water temperature, conductivity, PO_4_-P and TN/TP were best related to the spatiotemporal distribution of bacterial community, while water temperature, conductivity, NO_x_-N and transparency were closest related to the variation of eukaryotic community. Variation partitioning, based on partial RDA, revealed that environmental factors played the most important roles in structuring the microbial assemblages by explaining 11.3% of bacterial variation and 17.5% of eukaryotic variation. However, pure spatial factors (6.5% for bacteria and 9.6% for eukaryotes) and temporal factors (3.3% for bacteria and 5.5% for eukaryotes) also explained some variation in microbial distribution, thus inherent spatial and temporal variation of microbial assemblages should be considered when assessing the impact of environmental factors on microbial communities.

## Introduction

Microbes are mainly composed of bacteria, archaea, algae, protozoa, fungi and small metazoa, representing the most abundant and diverse group across ecosystems, and playing crucial roles in aquatic ecosystem functioning [1,2]. Microbes can be an important group of primary producers, can decompose organic matter and have unique capabilities in transforming nutrients along food webs in aquatic ecosystems [2]. In the past few decades, our knowledge on the spatiotemporal patterns of microbial abundance and production are well understood in oceans [1,3,4] and temperate lakes [5-7]. However, there are fewer studies focusing on patterns of microbial community composition in large rivers (e.g. hundreds of kilometers long) [8-10]. 

 In river ecosystems, microbial communities are driven by many interacting factors and processes, and it has been shown that environmental factors play the most important role in shaping the composition of microbial communities [11-13]. However, spatiotemporal variation in the distribution and abundance of microbes is an inherent property of ecological systems. Therefore, insufficient knowledge of microbial spatiotemporal variation can hinder the effective assessment of the relative importance of environmental factors in driving microbial community succession in structure and function [14]. A very powerful tool to address this issue is variation partitioning and ordination. Previous studies investigated microbial assemblages and their explanatory factors using variation partitioning and ordination procedures to partition the variation of species data into environmental and spatial components [15,16]. Here, we used a third matrix of explanatory variables that correspond to the temporal variation. Although partitioning in two independent matrices (eg. environmental and spatial matrices) has been carried out frequently in previous studies (see [15,16]), to the best of our knowledge, few microbial community fingerprinting studies published so far used the third matrix containing temporal variables. 

The Jiulong River is the second largest river in Fujian province, southeast China. It is an important drinking, agricultural and industrial water source for Longyan, Zhangzhou and Xiamen cities [17]. The upper stream of this river is one of the most important agricultural regions in Fujian. The excess input of nitrogen and phosphorus from intensive agricultural activities has significantly degraded the water quality in the upper Jiulong River in recent years. In a previous study, we investigated the composition of the microbial community in relation with agricultural and saltwater intrusion factors [12]. However, so far there is no information available concerning the seasonal change of microbial assemblages and their driving factors.

Thus, in this study we used denaturing gradient gel electrophoresis of PCR-amplified 16S and 18S rRNA gene fragments and multivariate statistical methods to explore the spatial (from the upper river to the estuary) and temporal (between dry and wet seasons) heterogeneity of planktonic bacterial and microbial eukaryotic communities in the Jiulong River. We were particularly interested in assessing the relative contribution of spatial, temporal and environmental factors affecting their composition. Our results showed that both bacterial and eukaryotic communities showed significant differences in composition over spatial and temporal scales in the river. Environment variables played the most important role in structuring microbial spatiotemporal patterns in the river, however, spatial and temporal factors also explained some variation in the composition of microbial communities.

## Materials and Methods

### Study area and sampling

The Jiulong River Watershed (116°46′55′′ - 118°02′17′′ E, 24°23′53′′ - 25°53′38′′ N) is located in southeast China. It is situated in the subtropical zone with monsoonal climate, which makes the river subject to seasonal changes in hydrology and aquatic environmental conditions. The rainfall is concentrated in spring and summer (wet season, April to September), while in autumn and winter (dry season, October to March) rainfall is much smaller in amount [17]. 

**Figure 1:**
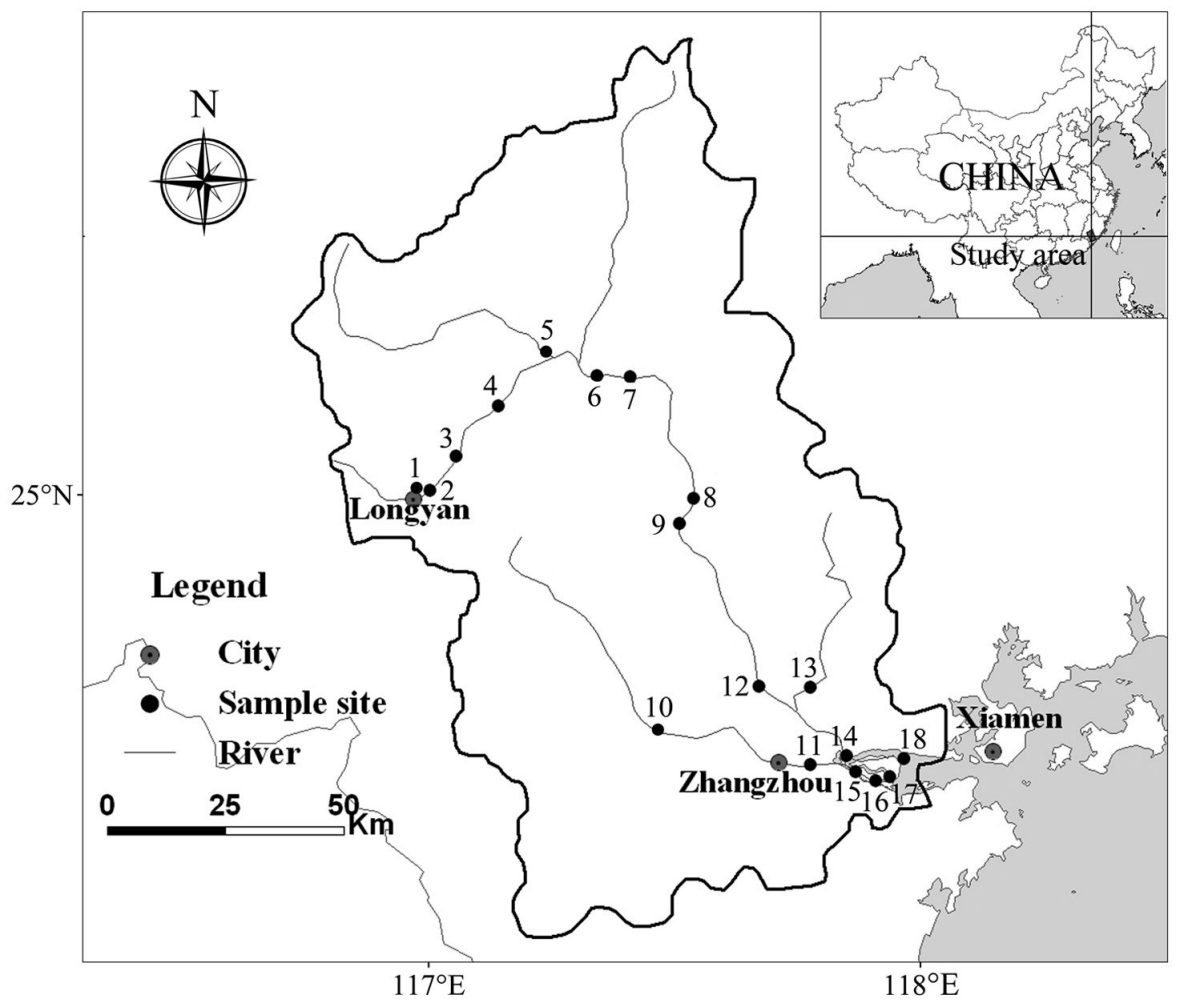
Location of sampling sites in the Jiulong River Watershed.

Surface water samples (2.5 L) were collected along the Jiulong River from eighteen sites in January and July 2010, respectively (Figure 1). Our sampling locations are not national park or other protected area of land. No specific permissions were required for our locations/activities and we confirm that the field studies did not involve endangered or protected species. Sites 1–4 were located at the upstream areas with intensive agricultural pollution (nitrogen and phosphorus). Sites 5–15 and sites 16–18 were located at the middle-lower Jiulong River and estuary, respectively. Water samples were transported to the laboratory as soon as possible. Microbial communities (500 mL water) for DGGE analysis were collected on 0.22 µm pore size polycarbonate filters (47 mm diameter, Millipore, USA). The water was pre-filtered through 200 µm mesh to remove the larger particles and metazoan. Then the filters were stored at -80°C until DNA extraction. 

### Environmental variables

Water temperature, conductivity, salinity, pH and dissolved oxygen (DO) were measured in situ with a Horiba W-23XD Multi-Parameter Water Quality Meter (Horiba, Japan). Water transparency was determined with a 30 cm Secchi disc. Suspended solids (SS) were determined gravimetrically by filtering 350 ml water sample through a pre-weighed filter (pore size of 0.45 µm), then weighing the filter again after drying at 105 °C. Total organic carbon (TOC) and total nitrogen (TN) were determined using a Shimadzu TOC-VCPH analyzer (Shimadzu, Japan). Total phosphorus (TP) was analyzed by spectrophotometry after digestion. Ammonium nitrogen (NH_4_-N), nitrite and nitrate nitrogen (NO_x_-N) and phosphate phosphorus (PO_4_-P) were measured with a Lachat QC8500 Flow Injection Analyzer (Lachat Instruments, USA).

### DNA extraction and PCR amplification

Total DNA was extracted directly from a 0.22-μm filter using an E.Z.N.A Soil DNA Kit (Omega Bio-Tek, USA) according to the manufacturer’s instructions. The DNA was quantified by spectrophotometer and stored at −40°C until further use.

The partial 16S and 18S rRNA genes were ampliﬁed by using the bacterial primers 341F-GC (5’-CGC CCG CCG CGC CCC GCG CCC GTC CCG CCG CCC CCG CCC GCC TAC GGG AGG CAG CAG-3’) [18] and 907R (5’-CCG TCA ATT CMT TTG AGT TT-3’) [19] and the eukaryotic primers Euk1A (5’-CTG GTT GAT CCT GCC AG-3’) and Euk516r-GC (5’-ACC AGA CTT GCC CTC CCG CCC GGG GCG CGC CCC GGG CGG GGC GGG GGC ACG GGG GG-3’) [20], respectively. The PCR mixtures (50 µl) contained 1 × PCR buffer, 1.5 mM MgCl_2_, 200 µM each deoxynucleoside triphosphate, 0.3 µM of each primer, 2.5 U of Taq DNA polymerase (TaKaRa, Japan), and approximately 40 ng of template DNA. The PCR program for bacterial primers began with a 5 min denaturation at 94°C; this was followed by 30 cycles of 94°C for 30 s, 52°C for 30 s, and 72°C for 60 s. The ﬁnal cycle was extended at 72°C for 10 min. The PCR program for eukaryotic primers began with an initial 130 s at 94°C; followed by 30 cycles of 30 s at 94°C, 45 s at 54°C, and 130 s at 72°C; followed by 10 min at 72°C. 

### DGGE

DGGE was performed with a DCode mutation detection system (Bio-Rad, USA) by using a 6% (w/v) polyacrylamide gel with a 30% to 60% gradient of a DNA-denaturant agent for separation of the 16S rRNA genes and 25% to 55% for the 18S rRNA genes, respectively. The 100% denaturant is deﬁned as 7 M urea and 40% (v/v) deionized formamide. For each sample, 800 ng of PCR product was loaded, and the electrophoresis was conducted at 100 V for 16 h at 60°C in 1×TAE buffer [19]. The gels were stained with SYBR Green I for 30 min, rinsed with distilled water, and then visualized under UV. DGGE patterns were analyzed using the Quantity One software (Bio-Rad, USA) as previously described [21]. Reproducibility was tested by replicate DGGE runs from all samples. The bands present in both replicate gels and the same position in the different lanes were identified and documented.

### Data analysis

The normality of the environmental variables were checked using Shapiro–Wilk test and variables were log(x+1) transformed with the exception of pH, to improve normality and homoscedasticity for multivariate statistical analyses. We used principal component analysis (PCA) to show main gradients in environmental variables. 

**Figure 2:**
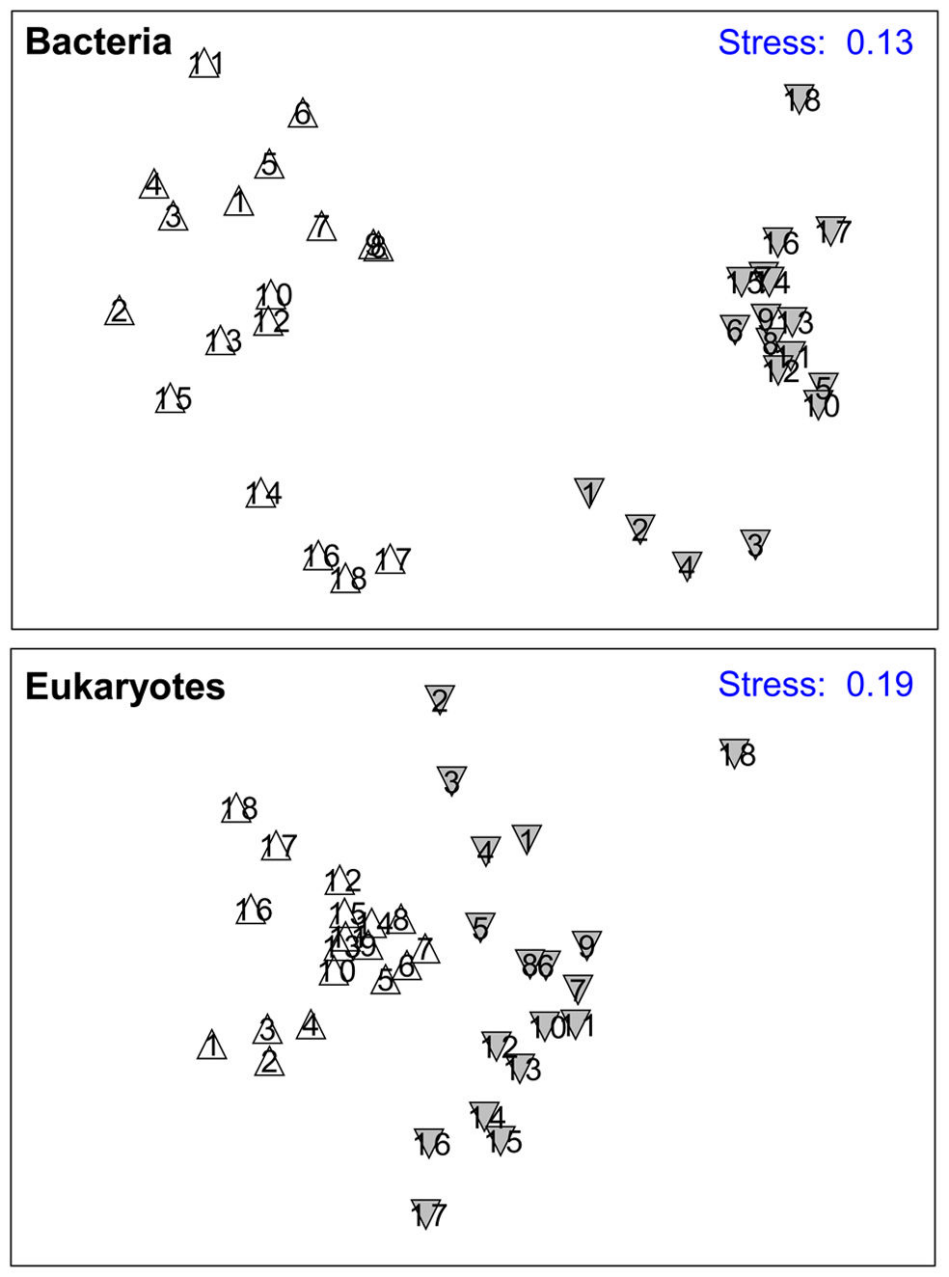
MDS ordination of DGGE fingerprints for bacterial and eukaryotic communities from the Jiulong River. The numbers indicate the sampling sites, which were collected in dry (△) and wet (▼) seasons, respectively.

**Figure 3:**
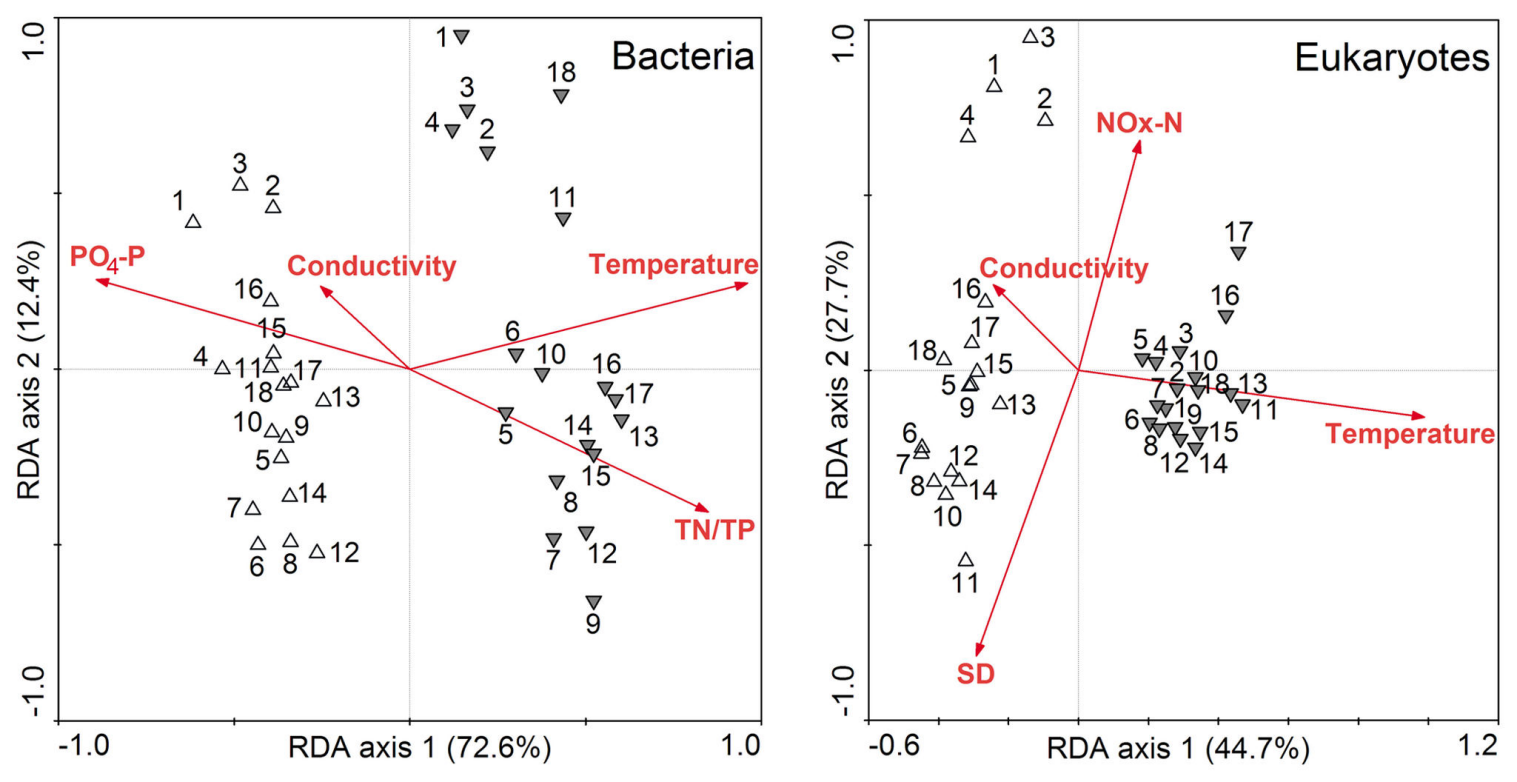
RDA ordination showing the microbial community composition in relation to significant environmental variables. The environmental variables were significantly related to the variation of microbial community composition (*P* < 0.05). The numbers indicate the sampling sites, which were collected in dry (△) and wet (▼) seasons, respectively.

**Figure 4:**
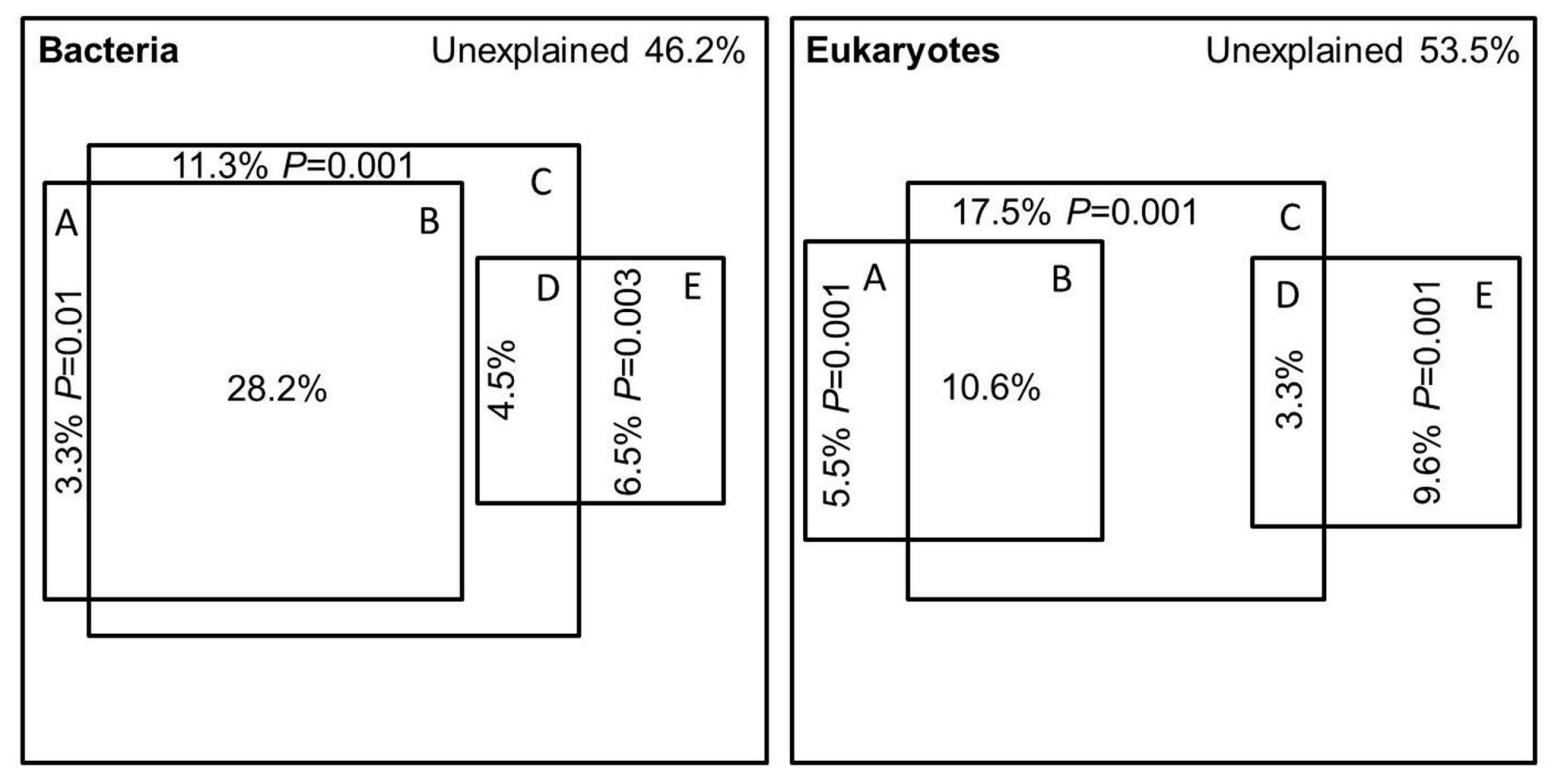
Variation partitioning between environmental, spatial and temporal variables. A = the pure temporal explanation; B = the temporal explanation that is shared by the environmental explanation; C = pure environmental explanation; D = the environmental explanation that is shared by the spatial explanation; E = pure spatial explanation.

We constructed two matrices for both bacterial and eukaryotic DGGE profiles, respectively. The first took into account the presence or absence of individual bands in all lanes (binary matrix), and the second contained the percentage of the intensity for each band based on the total intensity in the lane (intensity matrix). Statistical analyses were then performed based on the binary matrix (Figures 2, 3 and 4) and the intensity matrix (Figures S1, S2 and S3), respectively. However, the general trend of results obtained with these two matrices was similar. For these reasons, we only show the results obtained with binary matrix.

Bray-Curtis similarity matrices were constructed with the DGGE profiles generated from each site. The non-metric multidimensional scaling (MDS) ordination was used to investigate differences in microbial communities among sites [22]. To evaluate the significant differences between groups, we used the randomization/permutation procedure analysis of similarities (ANOSIM). The ANOSIM statistic R is calculated by the difference of the between-group and within-group mean rank similarities, thus it displays the degree of separation between groups. Complete separation is indicated by R = 1, whereas R = 0 suggests no separation [22]. 

Spearman rank correlations were used to determine the relationships between the similarity of microbial community composition (the Bray-Curtis similarity) and the geographic distance among samples.

Redundancy analysis (RDA) was performed to explore the relationships between microbial communities and environmental variables. This method was chosen because preliminary detrended correspondence analysis (DCA) on both bacterial community and eukaryotic community data revealed that the longest gradient lengths were shorter than 3.0, indicating that the majority of species exhibited linear responses to the environmental variation [23]. A forward selection procedure was first used to all environmental variables. To evaluate the significance of the conditional effects, Monte Carlo permutation of full models was applied with 999 unrestricted permutations [24]. 

A set of spatial variables were generated through the use of principal coordinates of neighbor matrices (PCNM) analysis [25,26] based on the longitude and latitude coordinates of each site. Further, a temporal matrix composed of two binary descriptive variables corresponding to the dry and wet seasons was used to represent the time. We used partial RDA as described by Anderson and Gribble [27] to partition the variation of the microbial data explained by environmental, spatial and temporal factors. To avoid artificially increasing the explanatory power in our analyses through the inclusion of redundant explanatory variables, we first ran a series of partial RDAs constrained to each individual environmental, spatial and temporal variable alone. Variables were only retained in the analyses if they explained a significant (*P* < 0.01) variation in the microbial data. All significance testing was assessed by Monte Carlo permutation tests using 999 unrestricted permutations in CANOCO version 4.5 [24]. Following the exclusion of all non-significant explanatory variables, we then ran three separate RDAs for all the significant environmental, spatial and temporal variables remaining. To eliminate collinearity among variables within each category, explanatory variables with the highest variance inflation factor (VIF) were sequentially removed until all VIFs were less than 20 [24]. Following this, we ran a forward-selection procedure to select the minimum set of environmental, spatial and temporal variables that could explain a significant amount (*P* < 0.01) of variation in the microbial data. Finally, four environmental (water temperature, conductivity, PO_4_-P and TN/TP), two spatial (PCNM1 and PCNM2) and two temporal (dry and wet seasons) variables were selected to perform bacterial variation partitioning. Similarly, four environmental (water temperature, conductivity, NO_x_-N, and transparency), two spatial (PCNM1 and PCNM2) and two temporal (dry and wet seasons) variables were selected to perform eukaryotic variation partitioning.

## Results

### Environmental characteristics

**Figure 5:**
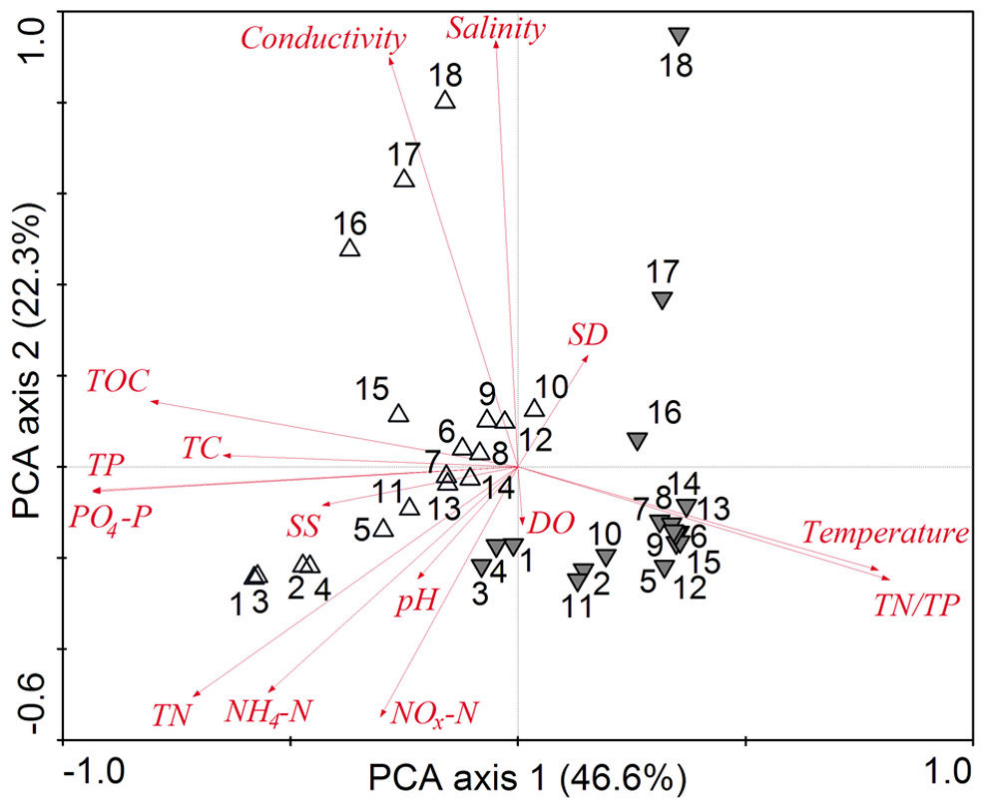
PCA plots showing the resemblance of environmental characteristics of sampling sites along the Jiulong River. The numbers indicate the sampling sites, which were collected in dry (△) and wet (▼) seasons, respectively.

Fifteen environmental variables in two seasons from 18 sampling sites are summarized in Table 1. The concentrations of TN, TP, PO_4_-P, TC and TOC were significantly higher in the dry season than in the wet season. In contrast, the temperature and TN/TP displayed significantly lower values in the dry season than in the wet season. PCA of 15 environmental parameters provided a clear distinction between the dry and wet seasons (Figure 5). The first two axes explained 68.9% of the total variability and effectively captured the main patterns of variation in the original variables. Along the first axis, variability was mainly explained by an increase in the TN/TP (r = 0.817, *P* < 0.01) and temperature (r = 0.793, *P* < 0.01) and a decrease in the PO_4_-P (r = –0.935, *P* < 0.01), TP (r = –0.935, *P* < 0.01), TOC (r = –0.807, *P* < 0.01) and TN (r = –0.715, *P* < 0.01). Variability along the second axis mainly corresponded to an increase in salinity (r = 0.936, *P* < 0.01) and conductivity (r = 0.900, *P* < 0.01) and a decrease in NO_x_-N (r = –0.550, *P* < 0.01).

**Table 1:** Physicochemical and biological parameters of the Jiulong River in the dry and wet seasons.

	Dry season (n = 18)			Wet season (n = 18)		*P*
Parameter	Mean	SD		Mean	SD	
Temperature (°C)	16.14	0.89		28.23	2.08	< 0.01
Transparency (m)	0.51	0.31		0.36	0.15	NS
Conductivity (μs cm^-1^)	5045	11608		2851	9460	NS
Salinity (‰)	2.95	7.32		1.69	6.02	NS
pH	7.29	0.44		7.26	0.27	NS
Suspended solids (mg l^-1^)	109.7	212.2		27.2	21.0	NS
DO (mg l^-1^)	7.75	1.63		7.51	0.98	NS
TC (mg l^-1^)	46.348	15.653		11.173	7.498	< 0.01
TOC (mg l^-1^)	32.037	14.672		1.827	1.013	< 0.01
TN (mg l^-1^)	7.831	5.200		4.376	2.275	< 0.05
NH_4_-N (mg l^-1^)	0.714	0.943		0.253	0.239	NS
NO_x_-N (mg l^-1^)	2.433	2.890		2.220	1.486	NS
TP (μg l^-1^)	448	526		60	67	< 0.01
PO_4_-P (μg l^-1^)	149	166		15	25	< 0.01
TN/TP mass ratio	25.88	12.20		135.16	90.05	< 0.01
Number of bacterial bands	19	2		27	3	< 0.01
Number of eukaryotic bands	42	5		25	4	< 0.01

n - Sample number; *P* - Level of significance (Independent-Samples T Test, *P* < 0.05) between the dry and wet seasons, NS – Not significant; SD - Standard deviation, DO - dissolved oxygen, TC - total carbon, TOC - total organic carbon, TN - total nitrogen, TP - total phosphorus.

### Microbial community

In total, 57 distinct bacterial bands were detected on the DGGE gels, the number of bacterial bands varied from 16 (site 3 in January) to 36 (site 18 in July) per sample (mean = 23, SD = 5, n = 36). The band number of eukaryotes was higher than that of bacteria (77 vs. 57) (Figure S4 and S5). The number of eukaryotic bands varied between 17 (site 18 in July) and 36 (site 6 in January) per sample (mean = 33, SD = 10, n = 36). The bacterial DGGE band number was significantly lower in dry season than in wet season. However, the number of eukaryotic bands was significantly higher in dry season than in wet season (Table 1). 

MDS ordination showed a clear separation of bacterial community composition as well as eukaryotic community composition between two seasons. However, both microbial communities separated into upper stream, middle-lower stream and estuary groups within seasons (Figure 2). In dry season, ANOSIM analysis revealed that the global *R* between upper stream, middle-lower stream and estuary groups was 0.577 at *P* = 0.001 for bacterial community and 0.869 at *P* = 0.001 for eukaryotic community. In wet season, the global *R* between upper stream, middle-lower stream and estuary groups was 0.950 at *P* = 0.001 for bacterial community and 0.812 at *P* = 0.001 for eukaryotic community, respectively. Also, the global *R* between two seasons was 0.946 at *P* = 0.001 for bacterial community and 0.488 at *P* = 0.001 for eukaryotic community, respectively. 

### Relationships between microbial communities and environmental factors

Forward-selection in RDA identified water temperature, conductivity, PO_4_-P and TN/TP were significant in explaining a large portion of the variation in bacterial community composition (*P* < 0.05), while water temperature, conductivity, NO_x_-N and transparency (SD) were significantly related to the variation of eukaryotic community composition (*P* < 0.05) in the Jiulong River (Figure 3). The cumulative variance of the species–environment relationship explained by the ﬁrst two RDAs were 85.0% in bacterial communities and 72.4% in eukaryotic communities, respectively.

### Correlation between the similarity of microbial community composition and geographic distance

 Spearman correlations revealed that the correlation coefficient was -0.371 (*P* < 0.01) between the similarity of bacterial community composition and geographic distance, while the coefficient of spearman correlation was -0.348 (*P* < 0.01) between the similarity of eukaryotic community composition and geographic distance.

### Variation partitioning

The partial RDA revealed that the environmental, spatial and temporal factors combined explained 53.8% and 46.5% of the total variation in bacterial and eukaryotic communities, respectively. The amount of variation explained by the pure environmental factors (11.3% for bacteria and 17.5% for eukaryotes) was the largest, followed by the pure spatial (6.5% for bacteria and 9.6% for eukaryotes) and the pure temporal (3.3% for bacteria and 5.5% for eukaryote) factors in both communities. More interestingly, 28.2% of the temporal variation and 4.5% of the spatial variation in bacterial communities were environmentally correlated, respectively, while 10.6% of the temporal variation and 3.3% of the spatial variation in eukaryotic communities were environmentally correlated, respectively (Figure 4). 

## Discussion

### Environmental factors driving the bacterial community

Our results showed that water temperature was strongly related to the seasonal variation of bacterial community composition. Field investigations in lacustrine and riverine ecosystems have demonstrated that water temperature covaried with the abundance and composition of bacterial community since each bacterial phylotype has its own optimal temperature range [28-30]. For example, Adams et al [31] showed that aquatic bacterial populations had different temperature optima in Arctic lakes and streams. At the optimal temperature, the bacterial activity was 2 to 11 fold higher than at other incubation temperatures. Consequently, abundance of each bacterial population probably changed as the water temperature ﬂuctuates, which resulted in variation of bacterial community composition in the Jiulong River. 

Nutrient concentrations and composition may have a significant influence on bacterial community composition because different bacterial organisms are adapted to different nutritional conditions [6,11]. Our results showed that the PO_4_-P and TN/TP explained the second largest portion of the variability in bacterial community composition. In fact, the discharge of excess nitrogen (N) and phosphorous (P) from agricultural activities and urban wastewater caused a serious water quality problem in the Jiulong River in recent decades [17]. Especially, during the dry season, the nutrients were concentrated due to the rare rainfall and low river flow. Hence, elevated concentration of nutrients may have contributed to changes in community composition via the direct inhibition of the microbes which were sensitive to environmental conditions, the increased dominance of microorganisms adapted to consume the N and P effectively, and their predator (i.e. ciliate) [32].

In the present study, the salinity increased gradually from site 16 to site 18, and the highest salinity was 27‰ in dry season site 18. The salinity strongly changed the bacterial community composition in Jiulong River estuary. In estuarine region, salinity is correlated highly with conductivity, and it is a major regulatory factor of aquatic bacterial communities [33,34]. Salinity inﬂuences the osmoregulation and metabolism of bacterial cells, such as the ability to assimilate different DOC compounds [35]. Our data suggest that estuarine bacteria are able to tolerate changes in salinity, which resulted in distinct change of bacterial community composition compared to the adjacent river [36]. Salinity also has a pronounced impact on the abundance and composition of zooplankton [37], which can strongly inﬂuence bacterioplankton diversity via top-down regulations.

### Environmental factors driving the eukaryotic community

In this study, RDA ordination revealed that water temperature was a key factor in regulating eukaryotic community composition. This result agrees with the observation of several studies that temperature can significantly influence the seasonal variation in microbial eukaryotic community composition [13,38,39]. As one of the main seasonal factors, water temperature may directly influence abundance and composition of eukaryotic communities. For example, some species may beneﬁt from warm temperature, since they have high optimum growth temperatures, while other species do not. Hence, the differences of water temperature between winter and summer in the Jiulong River could select different taxa by favoring the growth of some specific phylotypes, and thereby explaining variation of eukaryotic microbial communities.

NO_x_-N was the only nutrient that significantly related to the eukaryotic community composition in this study. NO_x_-N is an important form of inorganic nitrogen that can be taken up by phytoplankton, and every group of phytoplankton in different period has different ratio of NO_x_-N uptake [40,41]. In our study period, the concentration of NO_x_-N was higher, compared to the NH_4_-N, suggesting that the NO_x_-N might be a suitable nitrogen source for phytoplankton in the Jiulong River. Hence, NO_x_-N mediated the variation of phytoplankton community composition, and indirectly influenced the whole eukaryotic community composition in the river.

In this study, the conductivity increased quickly in the estuarine sites owing to the elevated salinity. Salinity has a strongly effect on abundance and composition of eukaryotic communities [42,43]. For example, Greenwald et al [37] assessed the effects of salinity on plankton assemblages, and demonstrated that the total zooplankton abundance decreased with salinity when salinities were above 17 ppt. In contrast, the total phytoplankton abundance increased with salinity, for salinities above 17 ppt. In our study, salinity was higher in the estuarine sites than those in the other sites, and three sites had salinity in excess of 17 ppt. Hence, the salinity was a primary influence on microbial eukaryotic communities in the Jiulong River estuary.

Transparency was correlated significantly with the eukaryotic community composition only. The RDA ordination revealed that the arrow of transparency point to the middle-low river sites. In the middle-low Jiulong River, the ﬂow of water was slower than that of the upstream sites, and the transparency increased with the sedimentation of the suspended solids. The low transparency at the upstream sites restricted the growth of phytoplankton because of light limitation. An increase in transparency may alleviate this light limitation and promote the growth of phytoplankton, with a resulting change in the composition of the eukaryotic communities in the middle-low river sites.

### Temporal and biogeographical patterns of the total microbial community

The explanation of pure temporal factors was fairly low (3.3% for bacterial and 5.5% for eukaryotic communities) indicating that the inherent temporal variation of microbial assemblages may be low in the Jiulong River. However, many of the environmental variables included in this study were temporally structured. This was not surprising, given that water temperature and nutrient concentrations were driven mainly by the seasonal climate and hydrology. In the Jiulong River Watershed, rainfall is concentrated in the spring and summer (from April to September), and the river flow is high during this period. On the contrary, rain events are scarce from October to January, characterizing a low river flow period in the region [17]. In our study, microbial communities in dry season were sampled during an extended period of low river flow, and the nutrients were significantly concentrated. In contrast, microbial communities in wet season were sampled during a high river flow period, which resulted in a dilution of the nutrients in the water column [44]. It appears that both climatic and hydrological conditions significantly influence the water physicochemical parameters and drive changes in the composition of microbial communities, thus suggesting a strong linkage between climate, hydrology, water quality and microbial communities. 

The spatial distribution and the distance between ecosystems might influence the dispersal of microbial cells and alter the aquatic microbial community composition [45-47]. There is some evidence that the spatial patterns of microbial dispersal clearly exist, and the microbial community composition is significant different when medium- (kilometers to tens of kilometers) to large- (hundreds to thousands of kilometers) scale data are analyzed [2,15,45,48]. Our analyses showed a robust relationship between the spatial configuration and microbial composition (*P* < 0.01, Figure 4), and the similarity of community composition decreased with increasing geographic distance. This suggests that spatial factors may be important in structuring assemblages and determining the degree of similarity between the different sites along the Jiulong River. In addition, the bacterial pure spatial explanation was lower than the eukaryotic pure spatial explanation. The possibility is that bacteria have better dispersal ability via water course, since they are smaller in size than the eukaryotic phytoplankton and zooplankton. Small and unicellular bacteria, without appendages and feeding apparatuses, are less susceptible to damage by turbulence and debris, when they transport through watercourses [49,50]. On the other hand, however, the transportation of zooplankton may be more limited, due to the physical damage and lower survivability by turbulence [51,52]. 

In conclusion, the microbial communities, both bacterial and eukaryotic, in the Jiulong River showed a similar and significant spatiotemporal change in composition during the study period. Most of the temporal variation in the composition of microbial communities was explained by the seasonal environmental variables such as water temperature, nutrients (PO_4_-P and TN/TP). However, the high concentration of NO_x_-N in upper stream, the high transparency in middle and low stream, and the high conductivity in estuary significantly influenced the spatially distribution of microbial communities. Although environment variables played the most important role in structuring microbial assemblages in the Jiulong River, pure spatial and temporal factors also explained some variation in the composition of microbial communities. Thus, inherent spatial and temporal variation of microbial assemblages should be considered when assessing the impact of environmental factors on microbial community at large spatial and temporal scales.

## Supporting Information

Figure S1 **MDS ordination of DGGE fingerprints for bacterial and eukaryotic communities from the Jiulong River.** The result was based on the DGGE relative intensity matrices. The numbers indicate the sampling sites, which were collected in dry (△) and wet (▼) seasons, respectively.
(TIF)

Figure S2 **RDA ordination showing the microbial community composition in relation to significant environmental variables.** The environmental variables were significantly related to the variation of microbial community composition (*P* < 0.05). The result was based on the DGGE relative intensity matrices. The numbers indicate the sampling sites, which were collected in dry (△) and wet (▼) seasons, respectively.
(TIF)

Figure S3 **Variation partitioning between environmental, spatial and temporal variables.** The result was based on the DGGE relative intensity matrices. A = the pure temporal explanation; B = the temporal explanation that is shared by the environmental explanation; C = pure environmental explanation; D = the environmental explanation that is shared by the spatial explanation; E = pure spatial explanation.
(TIF)

Figure S4 **DGGE profile of 16S rRNA gene fragments amplified from natural community in the Jiulong River.** Lanes 1-18 denote sampling sites 1-18, M - Marker.
(TIF)

Figure S5 **DGGE profile of 18S rRNA gene fragments amplified from natural community in the Jiulong River.** Lanes 1-18 denote sampling sites 1-18, M - Marker.
(TIF)
