# Supplementary figures and images for: Patterns in the Composition of Microbial Communities from a Subtropical River: Effects of Environmental, Spatial and Temporal Factors
Source: PLoS One. 2013 Nov 14;8(11):e81232. doi: 10.1371/journal.pone.0081232 (PMC3828266; doi:10.1371/journal.pone.0081232)

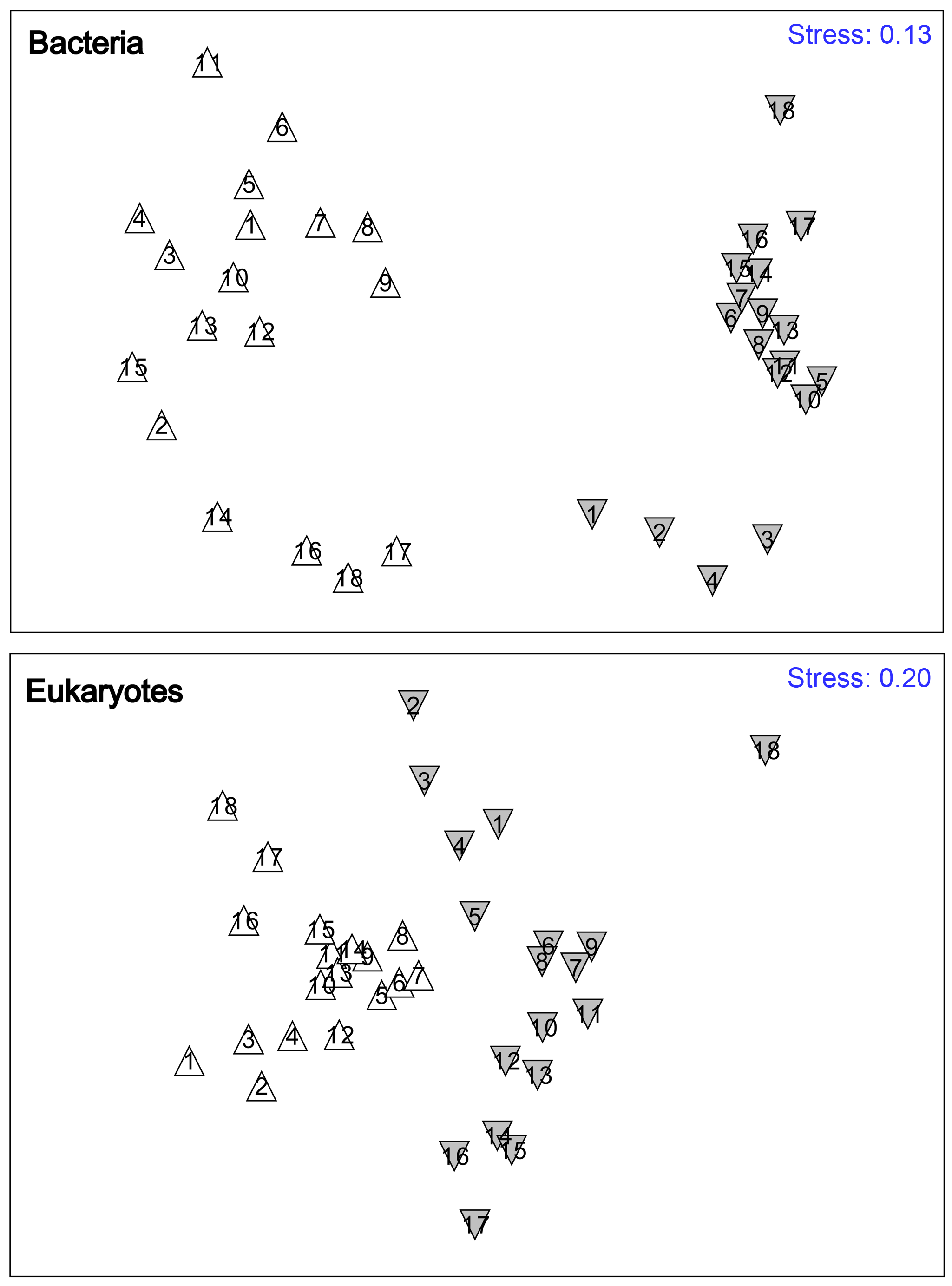

Supplement: Figure S1 — MDS ordination of DGGE fingerprints for bacterial and eukaryotic communities from the Jiulong River. The result was based on the DGGE relative intensity matrices. The numbers indicate the sampling sites, which were collected in dry (△) and wet (▼) seasons, respectively. (TIF) [file pone.0081232.s001.tif]

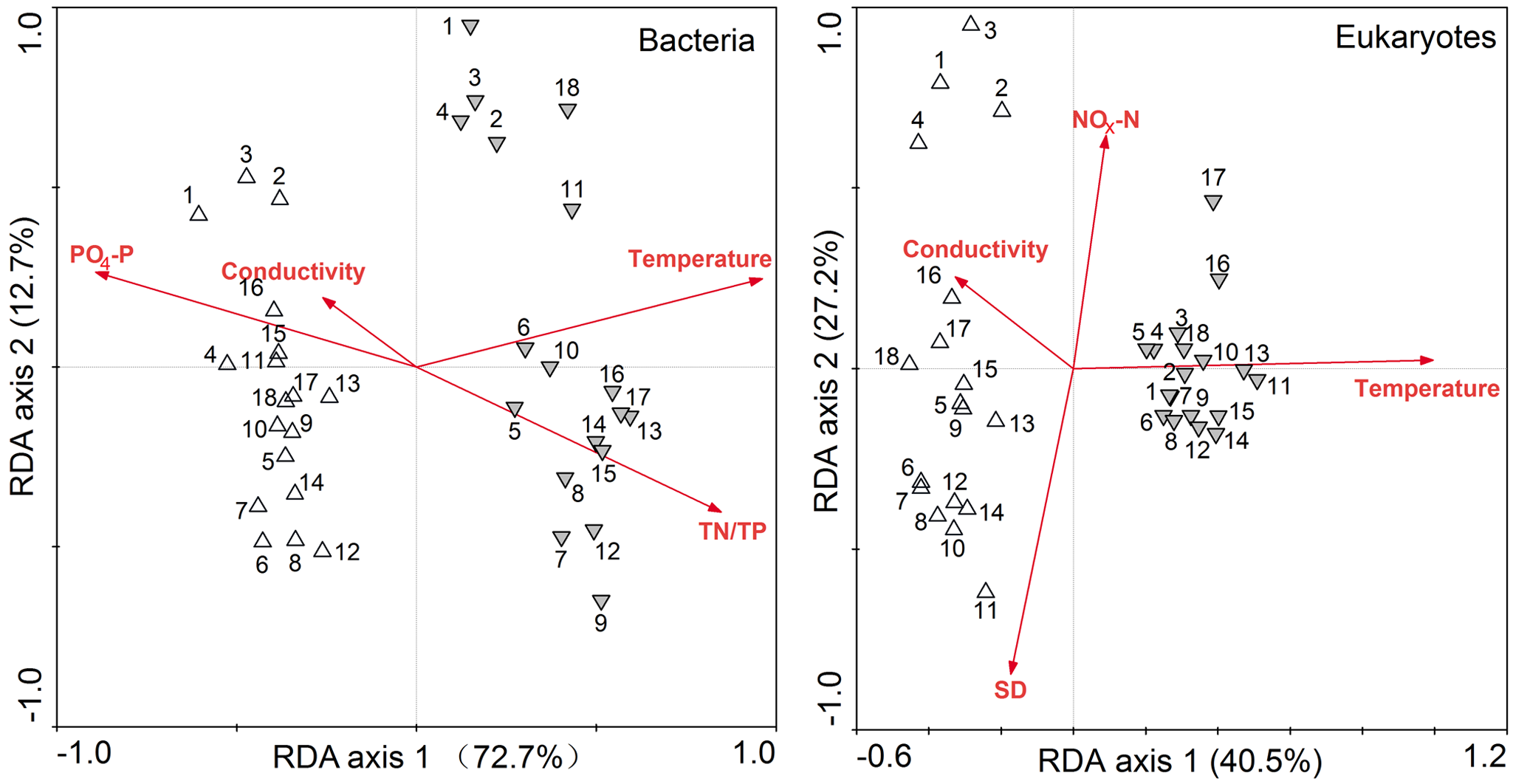

Supplement: Figure S2 — RDA ordination showing the microbial community composition in relation to significant environmental variables. The environmental variables were significantly related to the variation of microbial community composition (P < 0.05). The result was based on the DGGE relative intensity matrices. The numbers indicate the sampling sites, which were collected in dry (△) and wet (▼) seasons, respectively. (TIF) [file pone.0081232.s002.tif]

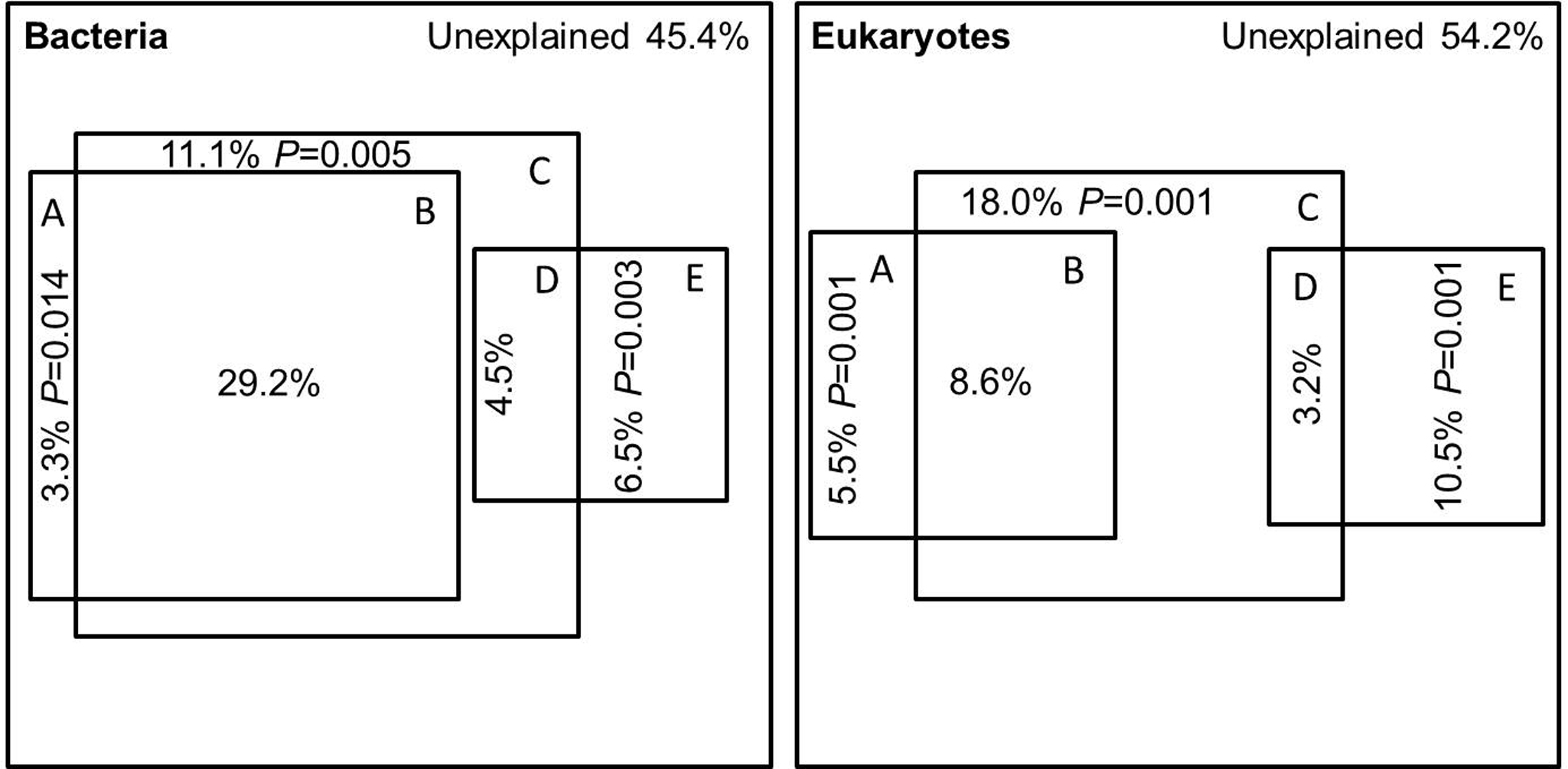

Supplement: Figure S3 — Variation partitioning between environmental, spatial and temporal variables. The result was based on the DGGE relative intensity matrices. A = the pure temporal explanation; B = the temporal explanation that is shared by the environmental explanation; C = pure environmental explanation; D = the environmental explanation that is shared by the spatial explanation; E = pure spatial explanation. (TIF) [file pone.0081232.s003.tif]

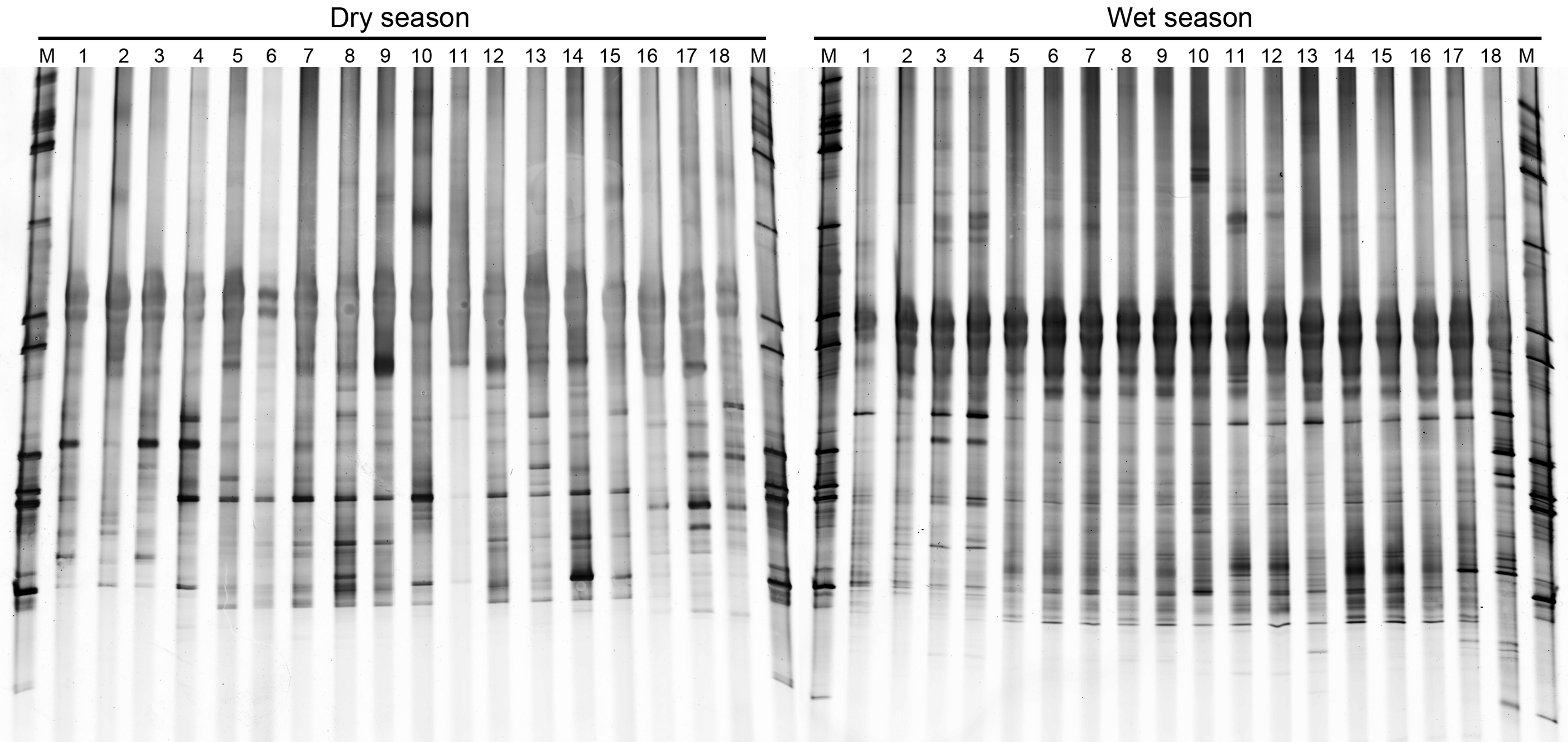

Supplement: Figure S4 — DGGE profile of 16S rRNA gene fragments amplified from natural community in the Jiulong River. Lanes 1-18 denote sampling sites 1-18, M - Marker. (TIF) [file pone.0081232.s004.tif]

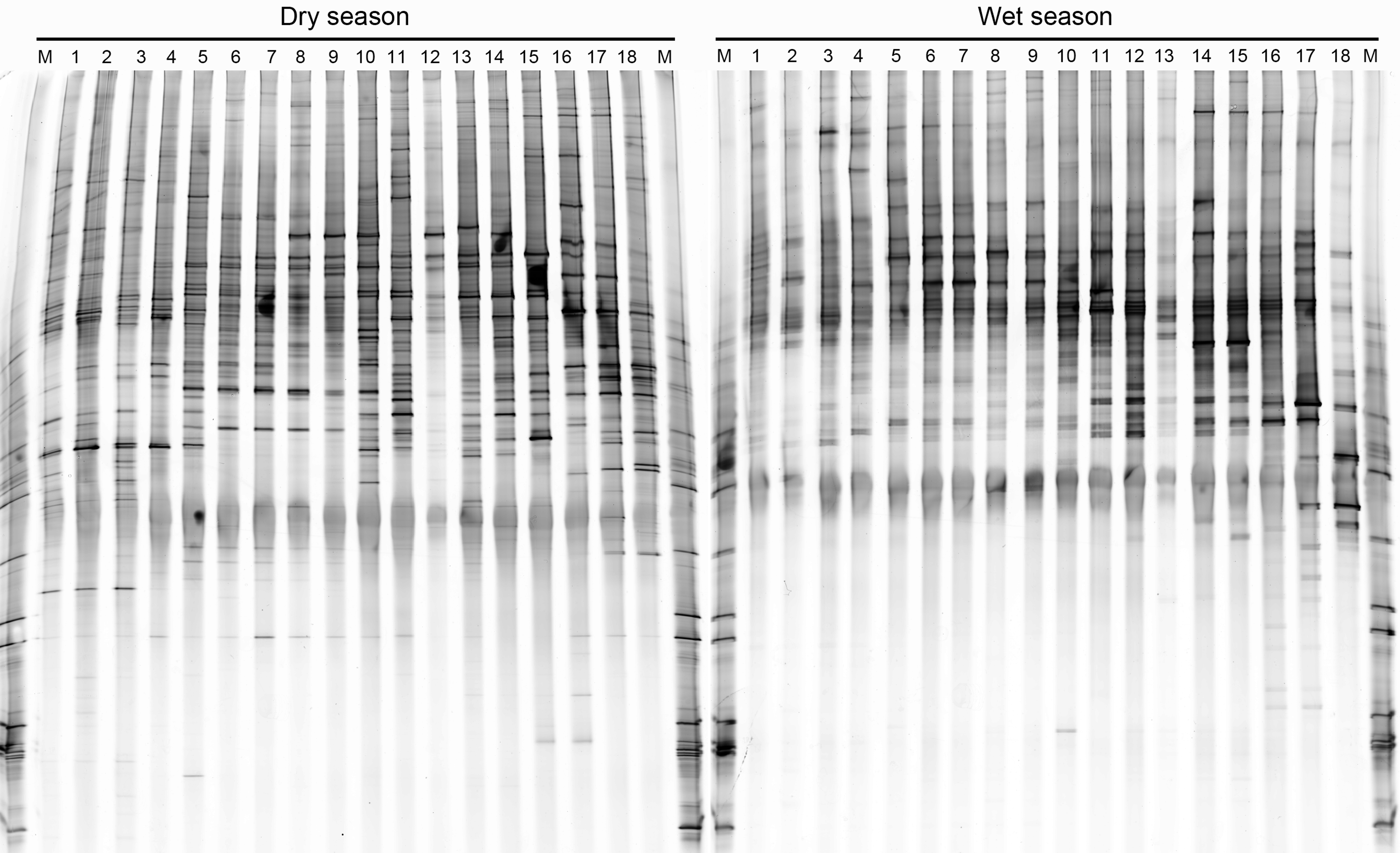

Supplement: Figure S5 — DGGE profile of 18S rRNA gene fragments amplified from natural community in the Jiulong River. Lanes 1-18 denote sampling sites 1-18, M - Marker. (TIF) [file pone.0081232.s005.tif]
